# Exploring soil bacterial diversity in different micro-vegetational habitats of Dachigam National Park in North-western Himalaya

**DOI:** 10.1038/s41598-023-30187-w

**Published:** 2023-02-22

**Authors:** Hina Mushtaq, Bashir Ahmad Ganai, Arshid Jehangir

**Affiliations:** 1 Terrestrial Ecology Laboratory, Department of Environmental Science, University of Kashmir, Hazratbal, Srinagar, Jammu and Kashmir 190006 India; 2 Centre of Research for Development, University of Kashmir, Hazratbal, Srinagar, Jammu and Kashmir 190006 India

**Keywords:** Ecology, Genetics, Microbiology, Molecular biology, Ecology, Environmental sciences

## Abstract

Dachigam National Park (DNP), in Zabarwan mountains of north-western Himalaya constitutes a region of high biodiversity with greater endemism. DNP is known for its unique micro-climate together with distinct vegetational zones providing home to variety of threatened and endemic plant, animal, and bird species. However, studies on soil microbial diversity in fragile ecosystems of north-western Himalaya in general and DNP in particular are lacking. This was thus a maiden attempt to study variations in soil bacterial diversity of DNP with respect to changing soil physico-chemical properties, vegetation, and altitude. Soil parameters depicted significant variations among different sites with highest values for temperature, OC, OM and TN being 22.2 ± 0.75 °C, 6.53 ± 0.32%, 11.25 ± 0.54%, 0.545 ± 0.04% from site-2 (low altitudinal grassland site) in summer and lowest of 5.1 ± 0.65 °C, 1.24 ± 0.26%, 2.14 ± 0.45% and 0.132 ± 0.04% at site-9 (high altitudinal mixed pine site) in winter. Bacterial CFU showed significant correlations with soil physico-chemical attributes. This study led to the isolation and identification of 92 morphologically varied bacteria with the highest (15) from site-2 and lowest (04) from site-9 which post BLAST analysis (via 16S rRNA analysis) depicted presence of only 57 distinct bacterial species under taxonomic phylum, Firmicutes and Proteobacteria. Nine species were widely spread (i.e., isolated from > 3 sites), however, most bacteria (37) were restricted to a particular site. Diversity indices ranged between 1.380 to 2.631 (Shannon–Weiner’s index); 0.747 to 0.923 (Simpson’s index) with highest values for site-2 and lowest for site-9. Index of similarity was highest (47.1%) between riverine sites (site-3 and site-4) whereas two mixed pine sites (site-9 and site-10) showed no similarity.

## Introduction

Bacteria are the most abundant and diverse group of organisms present in soil, catalyzing various life sustaining ecological processes on planet, Earth^1,2^. It is estimated that per gram of soil contains > 10^9^ microorganisms which represents around 4000–7000 genomes^3^. In soil, approximately 80–90% of soil processes are mediated by bacteria, as a result of which there is a greater interest in the relation between their diversity and function in the soil ecosystems^4^. Soil and its associated bacterial communities may be affected by different intertwined factors which may vary within the ecosystems thereby making the communities of bacteria distinctive to a particular ecosystem^5^. Therefore, keeping into consideration the contribution of bacterial populations in maintaining the ecological balance, as well as their flexibility to grow and adapt under varied physico-chemical conditions, cataloguing their diversity as it exists is of vital importance.

Among various soil microorganisms, bacteria are a major class which helps in keeping the soils healthy and fertile by their roles in cycling of nutrients like carbon, nitrogen, phosphorous and sulphur^1,6^. The bacteria in soil are known to perform vital services for the maintenance of the soil ecosystem health by improving the aggregation and structure of soil^7–9^, cycling of soil nutrients^1^, decomposition of organic matter^10,11^, enhancing soil fertility^12–14^, nitrogen fixation^15,16^, protection of plants against various pathogens^17^ etc. In addition, the bacteria in soil with their secretions bind to soil particles forming soil micro-aggregates that leads to the improvement of soil structure and quality thereby increasing the infiltration of water in soil that in turn enhance its water holding capacity^8^.

The soil bacterial population which is generally considered to be about one half of the total microbial biomass varying between 10^3^–10^6^ discrete genomes in a gram of soil typically depends on its physical, chemical and biological conditions^18,19^. Hence, soil bacterial community composition, structure and function relies on a variety of abiotic and biotic factors including physico-chemical characteristics of soil, nutrient availability, over-ground vegetation and ambient environmental factors^20–22^.

Among ecosystems, the supply of nutrients differs^23^, leading to variations in the community structure of plants and their production^24^. Typically, vegetation is known to influence and improve soil attributes like aeration, infiltration rate, hydraulic conductivity, structure, and water holding capacity^25^. However, bacterial diversity in soils has also been observed to be affected by seasonal fluctuations in vegetation leading to the replacement of dominant soil bacterial groups^26^.

Among various ecosystems, the Himalayas are known to inhabit great variety of soil microorganisms including mesophilic bacteria^27^. Earlier bacterial investigations in this part were limited to snow and glacier samples^28,29^ however, only recently focus has been shifted to assess the Himalayan soil bacterial diversity^30–35^. Specifically, the north-western part of Himalayas that encompasses through the erstwhile state of Jammu and Kashmir to Ladakh is considered to consist different climatic zones possessing characteristic attributes such as diverse soaring heights, alpine glaciers, lush green meadows, and a series of elevational zones having varied soil textures which inhabits the richest plethora of microorganisms particularly bacteria and actinomycetes having enormous biotechnological and bioprospecting potential^27,36^. Although there have been several attempts by various researchers for documenting soil bacterial diversity in this region^37–42^, only a few have focused on the forest ecosystems^43^ and protected areas (Table 1) while no study has been conducted in Dachigam National Park (DNP) which harbours various distinctive and endemic plant and animal biodiversity of the western Himalayan region^53–56^. Therefore, this study was taken up with a sole aim to generate the first ever baseline data on the culturable soil bacterial diversity, their altitudinal, seasonal and vegetational variations in different microhabitats of lower Dachigam National Park, Kashmir.

**Table 1:** Studies conducted on bacteria isolated from different soil and sediment samples in various protected areas of India.

Organism	Study area in India	Source	Culture dependent/independent	Medium of isolation	Estimation of colony forming units (CFU)	Number of strains identified	Mode of identification	Diversity assessment	References
Actinobacteria	NPs and WSs of Assam and Tripura	Soil	Dependent	Actinomycetes isolation agar	No	110	Conventional (morphological and physiological)	No	Thakur et al.^44^
Bacteria	WS in Rann of Kutch, Gujarat	Soil	Dependent	Complete medium broth (CMB)	No	15	Conventional (morphological, Gram staining and biochemical)	No	Rina et al.^45^
Bacteria	Dibru-Saikhowa NP and BR	Soil	Dependent	Nutrient agar	No	–	Conventional (morphological, Gram staining and biochemical)	No	Das et al.^46^
Bacteria	Murlen NP, Mizoram	Soil	Independent	Metagenomic DNA	–	302,416 sequences	Molecular (V4 region of 16S rRNA gene)	Yes	De Mandal et al.^47^
Bacteria	Multiple NPs in Gujrat	Soil	Dependent	Nutrient agar and Actinomycetes agar	Yes	–	Conventional (morphological and Gram staining)	Yes	Megha et al.^48^
Bacteria	Binsar WS, Uttarakhand	Soil	Dependent	–	Yes	–	Conventional (biochemical)	No	Kumar et al.^49^
Bacteria	Rajaji NP, Uttarakhand	Soil	Dependent	Nutrient agar	No	31	Conventional (biochemical)	Yes	Dhiman et al.^50^
Bacteria	Mangrove forest, Odisha	Sediment	Independent	Total DNA	–	6,42,505 sequences	Molecular (16S rRNA gene)	Yes	Behera et al.^51^
Actinobacteria	Pobitora WS and Kaziranga NP, Assam	Soil	Dependent	–	No	107	Molecular (16S rDNA-ARDRA)	No	Sharma and Thakur^52^
Bacteria	Dachigam NP, Kashmir Himalaya	Soil	Dependent	Nutrient, LB, and R2A agar	Yes	92	Molecular (16S rRNA gene)	Yes	Present study

*NP* National Park, *WS* Wildlife Sanctuary, *BR* Biosphere Reserve.

## Materials and methods

### Research area

**Figure 1:**
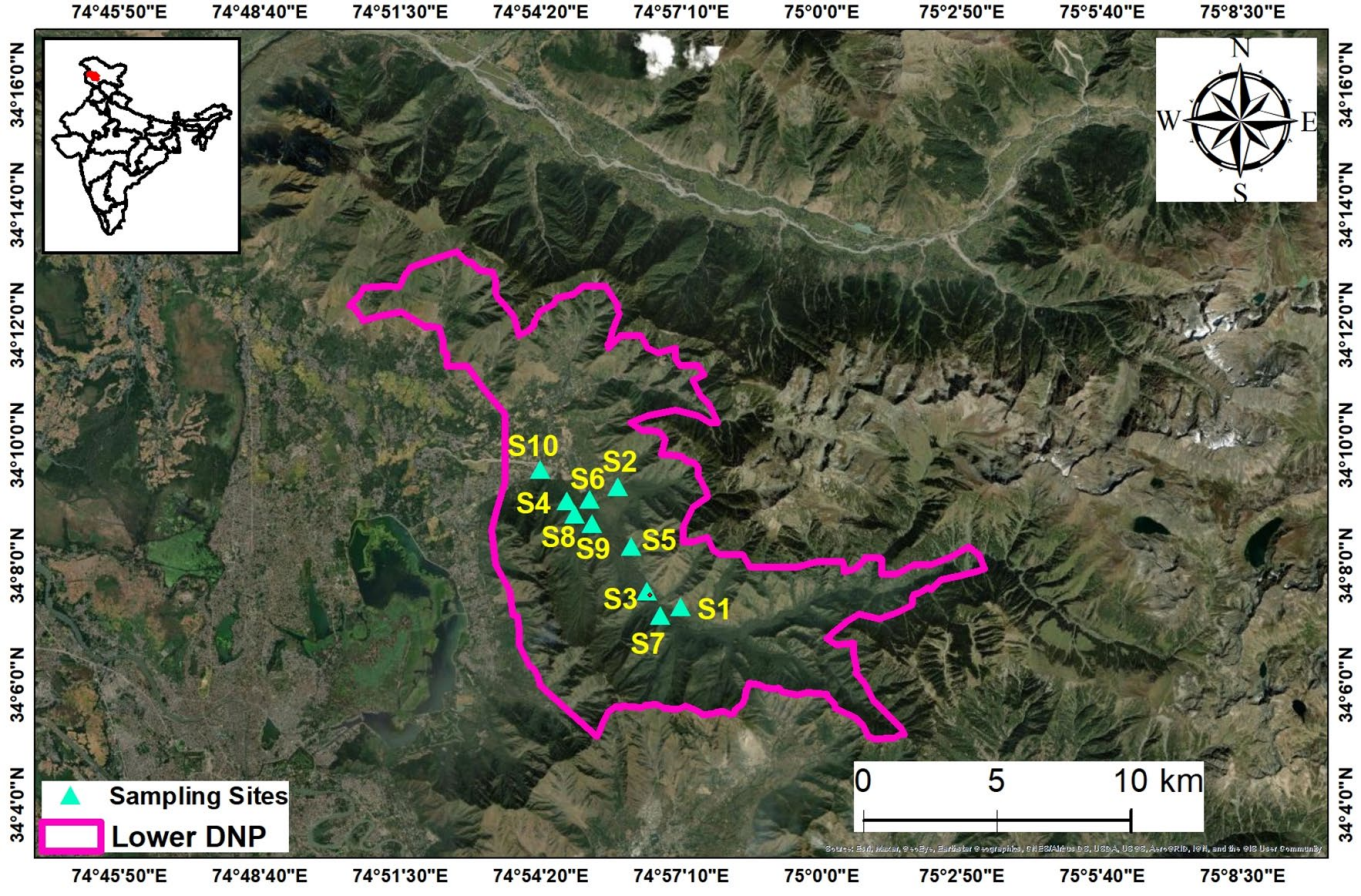
Map depicting different study sites in lower DNP. This figure was generated in ArcGIS version 10.4.1 (https://www.esri.com/en-us/arcgis/products/arcgis-pro/).

Dachigam National Park (henceforth referred to as DNP) located in the Zabarwan range of the Western Himalaya extends between 34°05′ N–34°11′ N and 74°54′ E–75°09′ E and stretches over 1677–4270 m altitude (Fig. 1). DNP roughly sprawls over 141 km^2^ officially comprising of two regions: lower Dachigam (26 km^2^) and upper Dachigam (115 km^2^) on the basis of altitude, forest types, and movement of its critically endangered red stag^57^. The current study was performed in lower DNP covering 1/3rd of its western end, and containing a deep gorge cut by Dagwan river (and its tributaries) originating in the Marsar lake (situated at about 4200 m altitude) of alpine Upper Dachigam (also called Dagwan Valley). Soils were sampled seasonally over a period of 2 years from ten different locations covering five different dominant vegetation types in lower DNP (Table 2), with each habitat type having a low and high altitudinal site so as to ascertain the influence of vegetation and altitude on the diversity of bacteria. The detailed vegetational attributes of each site is given in the table (Table 3).

**Table 2:** Characteristics of the selected study sites in Dachigam National Park, Kashmir.

Sites	Altitude in metres (amsl)	Latitude	Longitude	Dominant vegetation type
Site 1 High Altitudinal Grassland (HAG)	1870	34°07.373″N	74°56.870″E	Temperate Grassland
Site 2 Low Altitudinal Grassland (LAG)	1743	34°09.074″N	74°55.392″E	
Site 3 High Altitudinal Riverine (HAR)	1807	34°07.489″N	74°56.568″E	Mixed broad-leaved Riverine Vegetation
Site 4 Low Altitudinal Riverine (LAR)	1705	34°09.038″N	74°54.994″E	
Site 5 High Altitudinal Oak (HAO)	1770	34°08.304″N	74°55.963″E	Oak Vegetation
Site 6 Low Altitudinal Oak (LAO)	1733	34°09.090″N	74°55.320″E	
Site 7 High Altitudinal Parrotiopsis (HAP)	1832	34°07.405″N	74°56.695″E	Parrotiopsis Vegetation
Site 8 Low Altitudinal Parrotiopsis (LAP)	1710	34°08.961″N	74°55.013″E	
Site 9 High Altitudinal Mixed Pine (HAMP)	1743	34°08.792″N	74°55.226″E	Mixed Pine
Site 10 Low Altitudinal Mixed Pine (LAMP)	1671	34°09.468″N	74°54.339″E

**Table 3:** Vegetational characteristics of different study sites in lower Dachigam National Park, Kashmir.

Site	Vegetational attributes
HAG	*Stipa sibirica* with a few deciduous woody shrubs of *Cotoneaster nummularia, Indigofera heterantha,* having a xerophytic undergrowth mainly composed of *Agrimonia pilosa, Agrostis stolonifera, Alchemilla ypsilotoma, Anemone tschernjaewii, Artemisia absinthium, Bellis perennis, Bothriochloa ischaemum, Bromus arvensis, Bromus inermis, Bromus japonicus, Campanula latifolia, Capsella bursa-pastoris, Cirsium falconeri, Conyza canadensis, Cynodon dactylon, Cyperus rotundus, Dactylis glomerate, Daucus carota, Delphinium roylei, Euphorbia helioscopia, Galium aparine, Geranium nepalensis, Geranium pusillum, Lolium perenne, Medicago polymorpha, Nepeta cataria, Pennisetum orientale, Poa annua, Poa bulbosa, Poa pratensis, Ranunculus palmatifidus, Taraxacum officinale* and *Viola odorata*
LAG	*Themeda anathera* with the prevalence of *Celtis australis, Prunus armeniaca, Rubus niveus, Ulmus wallichiana* having an undergrowth of herbaceous plants like *Artemisia vestita, Colchicum luteum, Fragaria vesca, Lactuca dissecta, Origanum vulgare, Rosa webbiana, Rumex dentatus, Trifolium pratense, Trifolium repens* and *Verbascum thapsus*
HAR	*Acer caesium, Corylus *sp.,* Juglans regia, Populus alba, Populus cilia*, *Prunus cerasifera,* and *Rhus *sp., along with shrubs of *Berberis *sp.,* Robinia *sp., *Rubus ulmifolius, Rubus niveus, Prunus tomentosa,* and the undergrowth of *Bidens cernua*, *Dipsacus inermis, and Impatiens brachycentra*
LAR	*Prunus cerasifera, Morus alba, Morus nigra, Salix alba, Ulmus leavigata,* and *Ulmus wallichiana.* The shrub included various species of *Buddleja davidii, Indigofera heterantha, Rosa brunonii, Vibernum *sp., with a dominance of *Strobilanthes attenuate* while as the under-growth vegetation included species of *Alliaria *sp., *Clematis grata, Geranium *sp.,* Solenanthus circinatus,* and* Vitis vinifera*
HAO	*Quercus robur* with a predominance of tree species such as *Aesculus indica*, and *Morus alba,* shrubs like *Berberis lyceum, Indigofera heterantha, Isodon rugosus, Rosa webbiana* and herbs of *Artemisia nilagirica, Artemesia vestita, Chrysopogon echinulatus, and Origanum normale*
LAO	
HAP	*Parrotiopsis jacquemontiana,* and a few species of *Arthraxon lancifolius, Dipsacus mitis, Ziziphus anathera*
LAP	*Prunus armeniaca, Celtis australis, Ulmus wallichiana* and *Ulmus villosa.* Beneath the woody scrub of dominant tree species, *Parrotiopsis jacquemontiana*, there lied an undergrowth belonging to *Carex setigera, Dipsacus inermus, Fragaria vesca, Isodon rugusus, Lonicera quinquelocularis, Origanum normale, Rosa brunonii, Ziziphus pseudojujuba*
HAMP	*Pinus wallichiana,* with a pre-dominance of *Acer caesium, Parrotiopsis jacquemontiana, Picea smithiana* having a scarce vegetation of *Crataegus songarica, Lonicera quinquelocularis, Prunus cerasifera, Rhus succedanea, Rosa brunonii, Rosa webbiana, and Viburnum continifolium,* as well as herbaceous species of *Artemesia vestita, Cypripedium cordigerum, Dryopteris *spp.,* Geranium pratense, Polygonum amplexicaule, Origanum normale* and *Viola odorata*
LAMP	*Pinus wallichiana* having a scarce undergrowth of shrubs like *Berberis lyceum, Crataegus songarica, Geranium pratense, Lonicera quinquelocularis, Prunus cerasifera, Rhus succedanea, Rosa brunonii, Rosa webbiana, Viburnum continifolium and Viburnum grandiflorum*, and several other herbaceous plants belonging to species of *Arthraxon lancifolius, Aspidium *spp., *Hedera nepalensis, Polygonum* spp*., Viola indica, and Viola odorata*

### Soil sampling and sampling site parameters

Soils were sampled with a sterilized soil-corer up to a depth of 15 cm in poly-ethylene (PET) bags and sterile plastic vials were used for the assessment of the soil properties and bacterial analysis respectively^58^. At each site, five randomly collected soil samples were taken from different locations (approximately 8–10 m) on the same day and pooled together to get a composite representative sample for the site so as to document maximum diversity of the bacteria. The soil samples kept in sterile plastic vials were then stored at 4 °C until processed within 24 h^59^. Geographical coordinates of every sampling site were noted employing digital GPS (Garmin 7.6).

### Soil physico-chemical properties

Soil temperature was recorded on site at a depth of about 10–15 cm using a standard soil thermometer^58^. pH was determined by 1:2.5 (w/v) soil–water suspension using a digital pH meter^60^ and the percent moisture content in soil was analyzed gravimetrically^61^. The organic carbon and organic matter in soil was computed^62^ followed by the determination of soil total nitrogen by Kjeldahl method^63^.

### Isolation, enumeration and preservation of bacteria

The isolation of the culturable bacteria in the sampled soils was carried out by standard serial dilution and spread-plate method^64^. 1 g of each soil samples were put in 10 mL of sterile 0.85% NSS (normal saline solution), followed by thorough mixing in a shaking incubator for 4–5 min (120 rpm) so as to obtain a dilution series for inoculation. 100 μL (0.1 mL) aliquot from each dilution was gently spreaded on agar plates (Nutrient agar, Luria Bertani agar and Reasoner’s 2A agar) in triplicates. The agar plates were kept in incubation (24–48 h) at 37 ± 2 °C and the colonies which developed over the inoculated petri-plates were counted using digital Quebec-counter for assessing the soil bacterial colony forming units (cfu/g). The well-isolated colonies from each plate with different morphologies were then randomly selected and streaked onto the fresh agar plates. Pure isolates were maintained by re-streaking via sub-culturing and nutrient agar slants (stored at 4 °C) for future use^65^.

### Statistical analysis

In this study, all of the experiments were performed in triplicates and the results were expressed as Mean (± SD). Datasets have been subjected to Kruskal–Wallis test (p = < 0.0001), a non-parametric alternative to one-way ANOVA typically considered to be more appropriate than the traditional one-way ANOVA employing R-packages “tidyverse”, “ggpubr” and “rstatix”^66^. All possible pairwise comparisons were carried out by Wilcoxon’s test (p < 0.05) employing Dunn’s and Bonferroni adjustment.

### Correlation between soil physico-chemical properties and bacterial CFU

Correlation test, which measures the relationship between two or more variables was employed to determine the relation of bacterial colony-forming units with the soil properties. For the correlation analysis, Kendall’s rank-based correlation was used employing “ggpubr” package in R Software^66^.

### Identification of bacteria

The isolates were identified using morphological, Gram-staining and molecular approaches. The macro-morphological colony features of the isolated bacteria were assessed by Bergey′s manual^67^ followed by the Gram staining determination using Olympus 1X71 microscope. The 16S rRNA identification was carried out by extracting the DNA using QIAprep^®^ Spin Miniprep Kit (Catalog. No. 27104, by QIAGEN laboratories) following the manufacturers protocol with slight modifications. The extracted genomic DNA was utilized as a template for 16S rRNA gene amplification.

Amplification was performed by Polymerase Chain Reaction (PCR) in a thermo-cycler (CG Palm Cycler by Genetix Biotech Asia Pvt. Ltd) with universal bacterial primers^68^ synthesized by IDT (Integrated DNA Technologies) yielding a PCR product of about 1.5 kb. This was carried out in a final reaction mixture volume of 50 μL and the cycling parameters comprised of 5 min initial de-naturation (94 °C) followed by 30 cycles, each of de-naturation (94 °C) for 1 min, annealing (55 °C) for 45 s, extension (72 °C) for 2 min and final extension (72 °C) for 10 min. For negative control reaction, ultrapure (MilliQ) water was taken instead of exogenous template.

The amplicons of the expected size, approximately 1500 nucleotides (1.5 kb) were observed and confirmed via gel electrophoresis on 1.5% agarose gel in 1 × Tris–Acetate-EDTA (TAE) buffer with ethidium bromide stain wherein the banding patterns were visualized using UV illumination in a GEL DOC/Bio-imaging System. A 100 base pair DNA ladder (ThermoFischer SCIENTIFIC) was taken as a standard molecular weight DNA marker. The PCR products were sent to SciGenom Labs, Kerala for purification and subsequent partial DNA sequencing. Nucleotide Basic Local Alignment Search Tool (BLASTn) was used to identify all retrieved sequences by determining the phylogenetic neighbors from the databases of National Centre for Biotechnology Information (NCBI). Evolutionary phylogenetic trees were determined by the neighbor-joining method using Maximum Composite Likelihood as a correction factor by MEGA 7 with 1000 replicate bootstrap value.

### Assessment of soil bacterial diversity in DNP

The diversity of the isolated bacterial species community in terms of Shannon–Wiener’s (H′), Simpson’s index (d′), Dominance (D) and Evenness (*J*) was calculated using PAST 4.03 software^69^ whereas the similarity index of the bacterial species isolated from different study sites covering five dominant vegetational types located at varying altitudes was determined using Sorensen similarity index^70^.

## Results

### Soil physico-chemical properties

**Figure 2:**
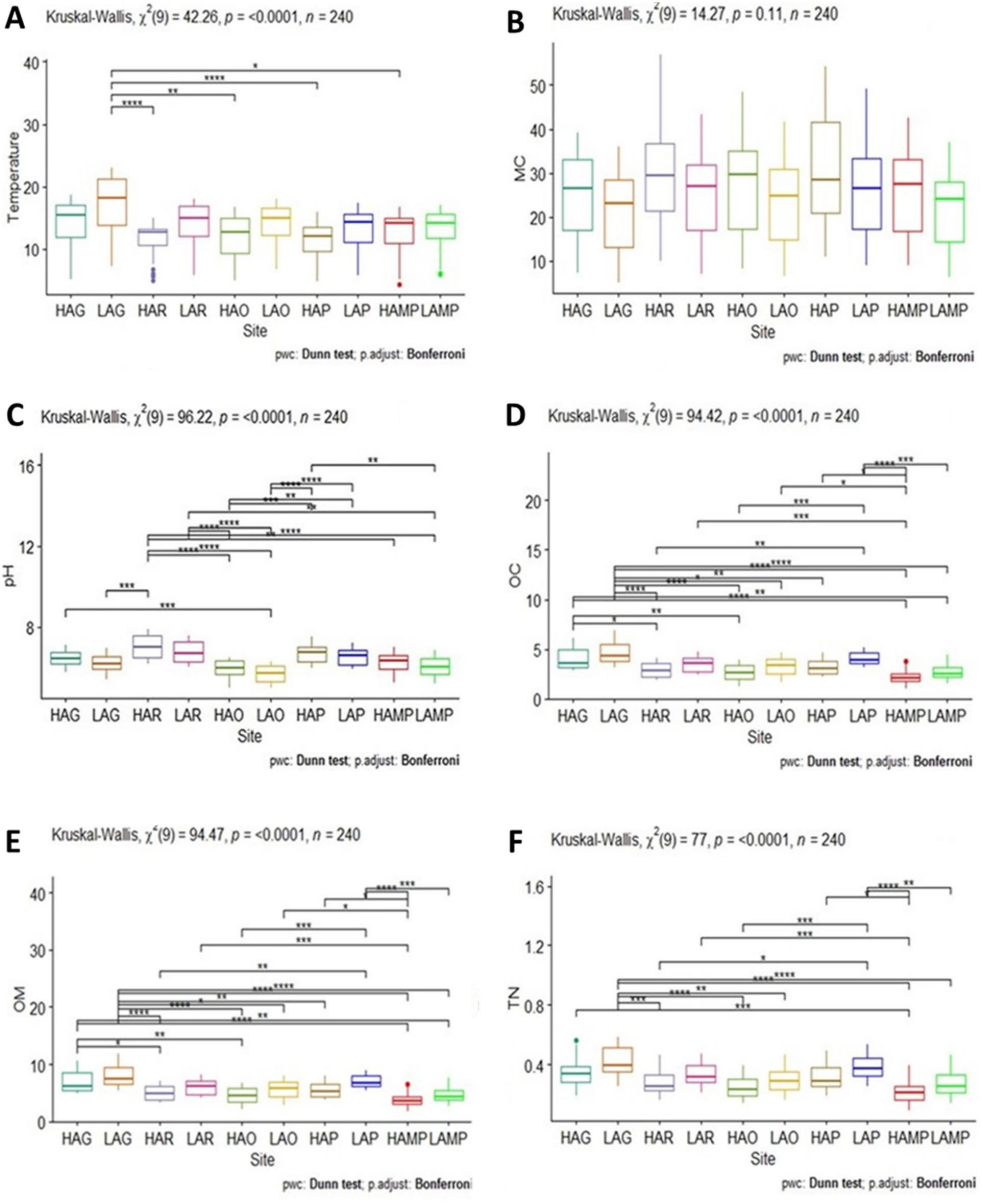
Mean site-wise variations in soil (**A**) temperature (°C), (**B**) moisture content (%), (**C**) pH, (**D**) organic carbon (%), (**E**) organic matter (%), and (**F**) total nitrogen (%) of lower DNP.

**Figure 3:**
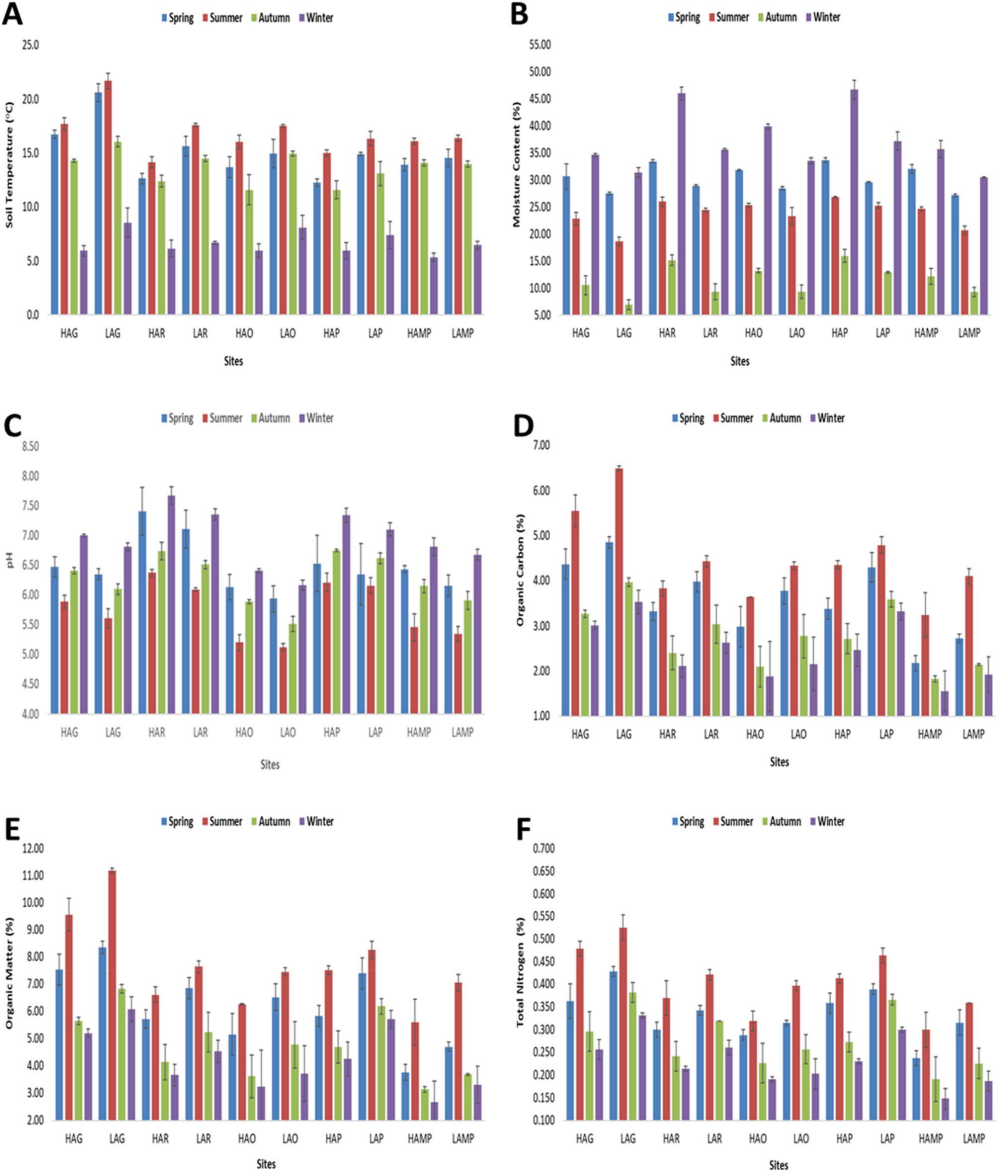
Mean seasonal variations in soil (**A**) temperature (°C), (**B**) moisture content (%), (**C**) pH, (**D**) organic carbon (%), (**E**) organic matter (%), and (**F**) total nitrogen (%) among the study sites in lower DNP.

Soil temperature depicted site-wise fluctuations with the lowest of 5.1 ± 0.65 °C recorded at site-9 (HAMP) in winter season and a highest of 22.2 ± 0.75 °C at site-2 (LAG) in summer. Statistically significant differences existed in the soil temperature (Fig. 2A) as suggested by Kruskal–Wallis test (p = < 0.0001) and Wilcoxon’s test (p < 0.05) whereby mean value of site-2 i.e., LAG (13.7 ± 4.76 °C) depicted highest significance with site-3 i.e., HAR (11.3 ± 3.20 °C), site-5 i.e., HAO (11.8 ± 3.90 °C) and site-7 i.e., HAP (11.2 ± 3.42 °C). In case of temperature, eta squared based on H statistic, displayed large effect (> 0.14) and about 14.5% variance was explained by the sites. Seasonally, soil temperature (Fig. 3A) ranged between 12.3 ± 0.4 to 20.6 ± 0.8 °C (spring), 14.2 ± 0.5 to 21.7 ± 0.7 °C (summer), 11.6 ± 0.8 to 16.1 ± 0.5 °C (autumn) and 5.4 ± 0.4 to 8.6 ± 1.3 °C (winter).

Soil moisture content (MC) which is the amount of water present within the soil was found to be highly variable among different study sites with the highest percent value of 47.87 ± 6.45% at site-7 (HAP) in winter and a lowest of 6.33 ± 1.15% at site-2 (LAG) during the autumn season. However, no statistically significant variations (p = 0.11) and effect size (eta squared) was observed across the sites (Fig. 2B). The seasonal range (Fig. 3B) of percent MC in soil was found to be 27.15 ± 0.2 to 33.60 ± 0.4% (spring), 18.65 ± 0.8 to 26.75 ± 0.2% (summer), 6.95 ± 0.9 to 15.95 ± 1.2% (autumn), and 30.45 ± 0.1 to 46.65 ± 1.8% (winter).

Soil pH displayed variations from one site to another wherein the highest pH of 7.78 ± 0.12 and a lowest of 5.11 ± 0.10 was noted from site-3 (HAR) in winter and site-6 (LAO) in summer season respectively. Significant difference existed between all sites (Fig. 2C) with the site depicting highest mean pH of 7.05 ± 0.57 i.e., site-3 (HAR) showing greater differences with site-5 (HAO, 5.91 ± 0.47), site-6 (LAO, 5.68 ± 0.42), site-9 (HAMP, mean pH 6.21 ± 0.52) and site-10 (LAMP, mean pH 6.02 ± 0.51). Moreover, 37.9% of variance was explained by the sites, with respect to pH. Seasonally, the soil pH (Fig. 3C) ranged between 5.94 ± 0.22 to 7.41 ± 0.40 (spring), 5.13 ± 0.06 to 6.37 ± 0.06 (summer), 5.52 ± 0.13 to 6.75 ± 0.02 (autumn) and 6.16 ± 0.08 to 7.68 ± 0.15 (winter).

The percent soil organic carbon (SOC) varied between the sites wherein the lowest (1.24 ± 0.26%) and highest (6.53 ± 0.32%) OC levels were found at site-9 (HAMP) in winter and at site-2 (LAG) in summer season respectively. Between the sites (Fig. 2D), the mean SOC values of site-2 (LAG, 4.71 ± 1.19%) were found to show higher statistical significance with site-3 (HAR, 2.92 ± 0.74%), site-5 (HAO, 2.65 ± 0.82%), site-9 (HAMP, 2.20 ± 0.71%), and site-10 (LAMP, 2.72 ± 0.89%). Moreover, the amount variance in SOC explained by site variation was regarded large (37.1%). The seasonal range of soil OC (Fig. 3D) was 2.18 ± 0.17 to 4.85 ± 0.13% (spring), 3.25 ± 0.49 to 6.49 ± 0.06% (summer), 1.83 ± 0.06 to 3.97 ± 0.09% (autumn) and 1.56 ± 0.45 to 3.54 ± 0.26% (winter). As soil organic matter (SOM) is determined from SOC, it also exhibited the same trend between the sites and seasons with the lowest of 2.14 ± 0.45% reported from site-9 (HAMP) in winter and a highest of 11.25 ± 0.54% from site-2 (LAG) in summer season respectively. Average SOM values also were found to have higher statistically significant differences among site-2 (LAG, 8.12 ± 2.05%) and site-3 (HAR, 5.03 ± 1.28%), site-5 (HAO, 4.57 ± 1.42%), site-9 (HAMP, 3.79 ± 1.22%), and site-10 (LAMP, 4.69 ± 1.54%) with the amount variance being large (37.2%) as explained by site variation based on eta squared values (Fig. 2E). Seasonally (Fig. 3E), the percent SOM ranged between 3.77 ± 0.29 to 8.36 ± 0.23% (spring), 5.60 ± 0.84 to 11.19 ± 0.09% (summer), 3.14 ± 0.11 to 6.84 ± 0.16% (autumn) and 2.69 ± 0.77 to 6.09 ± 0.45% (winter).

The disparities in the soil total nitrogen content were observed among the sites, with the highest of 0.545 ± 0.04% recorded at site-2 (LAG) in summer and a lowest of 0.132 ± 0.04% noted from site-9 (HAMP) in winter season. Mean values of nitrogen at site-2 (LAG, 0.416 ± 0.09%) displayed greater statistically significant difference with site-5 (HAO, 0.256 ± 0.08%), site-9 (HAMP, 0.219 ± 0.07%) and site-10 (LAMP, 0.271 ± 0.09%) as per Kruskal–Wallis test (p = < 0.0001) and the Wilcoxon’s test (p < 0.05). Moreover, the amount variance in nitrogen as explained by the site variation based on eta squared values was 29.6% which is often regarded large (Fig. 2F). The seasonal variation in the percent soil nitrogen (Fig. 3F) ranged between 0.238 ± 0.02 to 0.428 ± 0.01% (spring), 0.300 ± 0.04 to 0.526 ± 0.03% (summer), 0.191 ± 0.05 to 0.382 ± 0.02% (autumn) and 0.148 ± 0.02 to 0.331 ± 0.01% (winter).

### Enumeration of bacteria

Seasonal fluctuations in the bacterial colony forming units were observed with the CFU per soil gram increasing from spring to summer seasons followed by a decrease in autumn seasons and the lowest being recorded in the winter seasons. On comparatively analyzing the sites, altitudinal differences were also noted wherein sites located at low altitude had more bacterial colony count than the ones at the higher altitude and this was a trend present among all the study sites (high as well as low altitudinal sites) based on the five vegetation types. Subsequently bacterial density as CFU/g of soil was the highest (2.98 ± 0.03 × 10^−7^) at site-2 which is the low altitudinal Grassland site (LAG) being recorded during year 2 of study in summers whereas the lowest (1.23 ± 0.04 × 10^−7^) was observed in winter season of first sampling year at site-9 being the high altitudinal Mixed Pine, (HAMP) site (Table 4).

**Table 4:** Yearly seasonal variations in soil bacterial colony forming units (CFU) obtained from selected study sites in Dachigam National Park, Kashmir.

Sites	Mean CFU/g of soil (× 10^7^)							
	Year 1				Year 2			
	Spring	Summer	Autumn	Winter	Spring	Summer	Autumn	Winter
HAG	2.62 ± 0.04	2.80 ± 0.04	2.12 ± 0.03	1.36 ± 0.03	2.68 ± 0.03	2.88 ± 0.04	2.17 ± 0.04	1.39 ± 0.04
LAG	2.79 ± 0.05	2.97 ± 0.03	2.17 ± 0.04	1.38 ± 0.04	2.83 ± 0.04	2.98 ± 0.03	2.24 ± 0.04	1.43 ± 0.04
HAR	2.72 ± 0.02	2.91 ± 0.02	2.14 ± 0.04	1.39 ± 0.04	2.74 ± 0.05	2.94 ± 0.04	2.20 ± 0.04	1.46 ± 0.04
LAR	2.76 ± 0.03	2.94 ± 0.02	2.19 ± 0.05	1.46 ± 0.04	2.80 ± 0.04	2.95 ± 0.03	2.23 ± 0.02	1.48 ± 0.03
HAO	2.69 ± 0.03	2.91 ± 0.03	2.16 ± 0.04	1.40 ± 0.03	2.74 ± 0.04	2.93 ± 0.04	2.21 ± 0.03	1.46 ± 0.03
LAO	2.72 ± 0.04	2.93 ± 0.02	2.17 ± 0.04	1.44 ± 0.03	2.78 ± 0.03	2.95 ± 0.03	2.25 ± 0.05	1.49 ± 0.05
HAP	2.54 ± 0.04	2.71 ± 0.03	2.09 ± 0.04	1.33 ± 0.03	2.59 ± 0.03	2.75 ± 0.03	2.11 ± 0.04	1.39 ± 0.02
LAP	2.58 ± 0.04	2.82 ± 0.03	2.11 ± 0.03	1.34 ± 0.03	2.65 ± 0.04	2.79 ± 0.04	2.15 ± 0.03	1.41 ± 0.04
HAMP	2.50 ± 0.02	2.72 ± 0.06	2.02 ± 0.03	1.23 ± 0.04	2.55 ± 0.06	2.74 ± 0.05	2.07 ± 0.02	1.28 ± 0.05
LAMP	2.51 ± 0.01	2.78 ± 0.05	2.06 ± 0.03	1.29 ± 0.05	2.60 ± 0.03	2.76 ± 0.05	2.09 ± 0.03	1.31 ± 0.03

**Figure 4:**
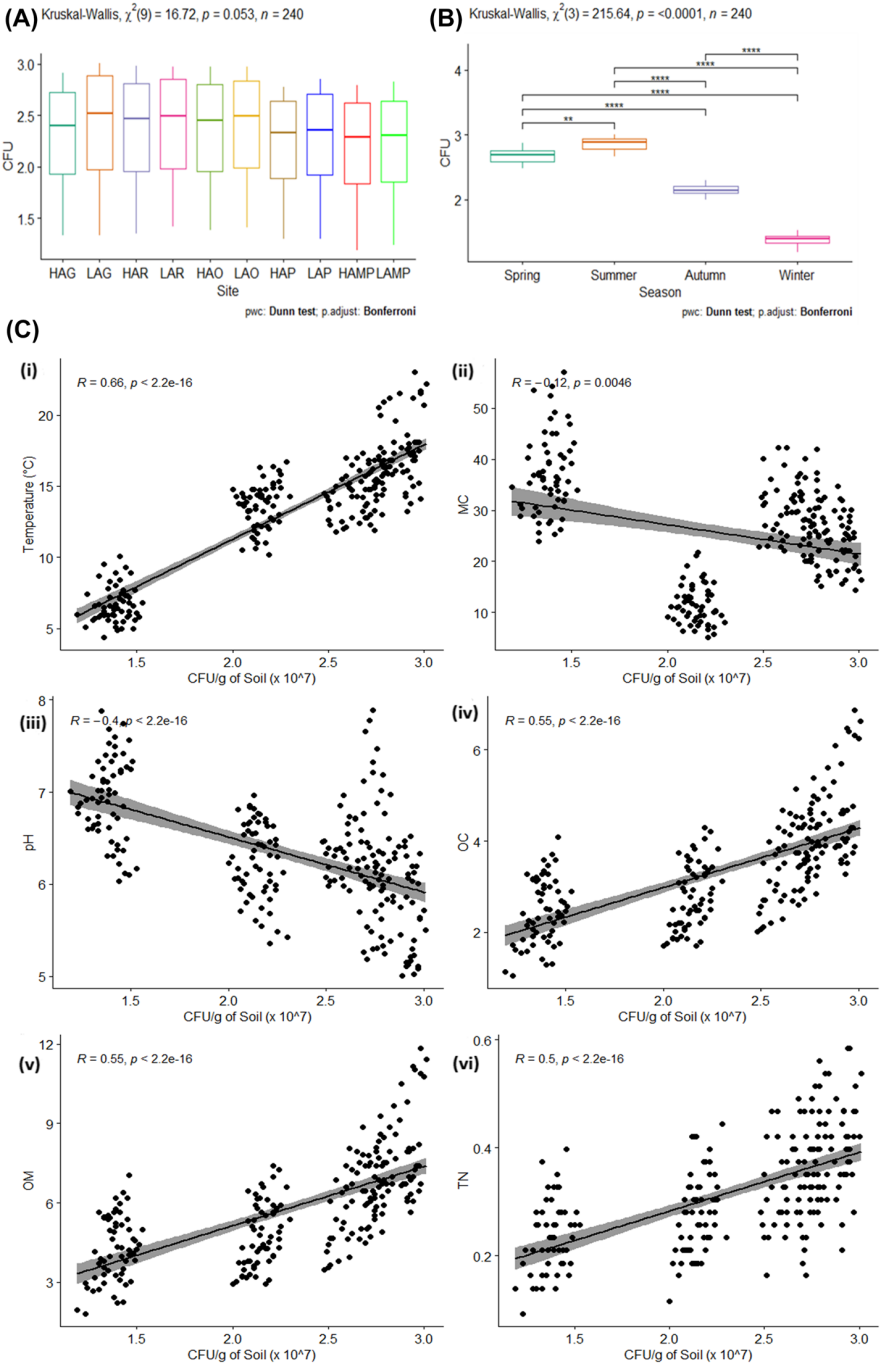

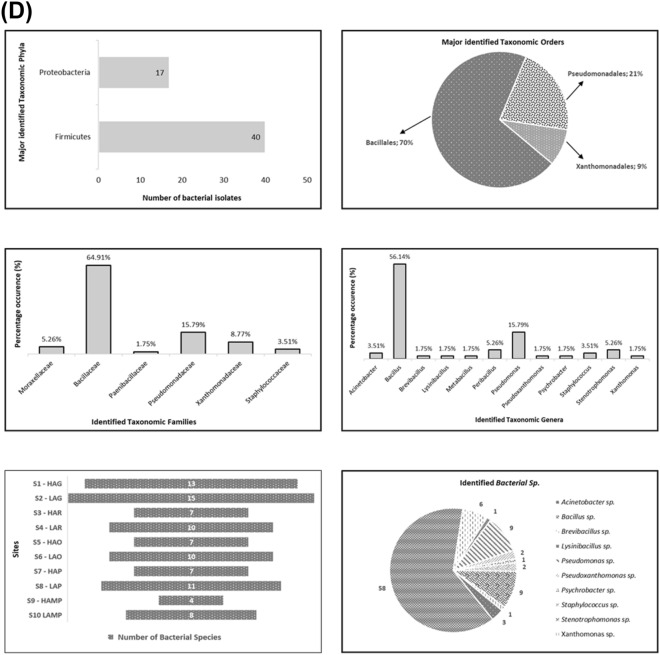
Site-wise (**A**) and seasonal variations (**B**) in soil bacterial colony forming units (CFU) of lower DNP. (**C**) Correlation of bacterial colony forming units (CFU) with various physico-chemical properties of soil—(**i**) temperature, (**ii**) moisture content, (**iii**) pH, (**iv**) organic carbon, (**v**) organic matter, and (**vi**) total nitrogen obtained from different micro-vegetational habitats of lower DNP. (**D**) Taxonomic classification of bacterial strains isolated from Dachigam National Park, Kashmir.

Statistically, no significant differences were noticed between the sites with respect to CFU using Kruskal–Wallis test (p = < 0.0001), and pairwise Wilcoxon’s test (Wilcoxon’s test, p > 0.05). Furthermore, the eta squared based on H statistic, suggested that in case of CFU, no large effect (< 0.036) was displayed as about 3.6% variance was explained by the sites (Fig. 4A). However, during this study, statistically significant differences with respect to CFU were observed among various seasons as determined by Kruskal–Wallis test (p = < 0.0001) and pairwise Wilcoxon’s test (p < 0.05). The eta squared based on H statistic, revealed that in case of seasonal colony-forming units (CFU), large effect (> 0.90) was displayed and about 90% variance in CFU was explained by the seasons (Fig. 4B).

### Correlational study of soil bacterial CFU with soil physico-chemical properties

During the 2-year study period, the results of the correlations tests between the bacterial colony-forming units (CFU) and various soil physico-chemical properties, suggested that their correlations were statistically significant (Fig. 4C). Bacterial CFU depicted significant positive correlations with soil characteristics like temperature (r = 0.66), organic carbon (r = 0.55), organic matter (r = 0.55) and total nitrogen (r = 0.50) whereas significant negative correlations were displayed with moisture content (r = − 0.12) and pH (r = − 0.4) of soil, with p values for all the tested parameters being less than the significance level alpha (= 0.05).

### Identification of bacteria

On the basis of morphological identification, a total of 92 morphologically different bacteria were isolated, of which the maximum isolates (15) were obtained for low altitudinal Grassland site (LAG) i.e., site-2 while as the minimum number of isolates (04) were recorded for the high altitudinal Mixed Pine, (HAMP) i.e., site-9 (Fig. S1A). All the isolated strains of bacteria depicted marked variations in macro-morphological attributes of their colonies, the detailed identification of which are given in the table (Table S1). The results of Gram staining suggested the dominance of Gram-positive (64.13%) followed by Gram-negative (23.91%) rod-shaped bacterial forms (*Bacilli*), existing either as a single, diplo-bacilli, or short/long chains. The presence of Gram-positive (6.52%) and Gram-negative (5.43%) round-shaped *Cocci* forms on the other hand were much less prevalent and arranged as cocci, diplo-cocci, or simply as bacterial clusters (Table S2).

The retrieved nucleotide sequences of the bacterial strains that were isolated from the ten (10) sampling sites in lower DNP and identified through 16S rRNA gene approach (Fig. S1B), which is a greatly conserved gene typically used for the prokaryotic species identification, were blasted (BLASTn) in the available databases of NCBI so as to find their closest neighbor. After proper percent similarity check and identification of all isolated bacterial strains, they were deposited in GenBank, NCBI and their accession numbers were availed (Table S3).

During this study, out of the total ninety-two (92) identified bacterial species, only fifty-seven (57) were found to be taxonomically different at a species level after combining all varied strains of a particular bacterial species (Fig. 4D). The overall systematic diversity of the isolated bacterial strains reflected the presence of two major taxonomic phyla of soil bacteria i.e., Firmicutes (40 species) and Proteobacteria (17 species) covering two classes (Bacilli and Gammaproteobacteria), three orders (Bacillales, Pseudomonadales and Xanthomonadales), six families (Bacillaceae, Paenibacillaceae, Staphylococcaceae, Moraxellaceae, Pseudomonadaceae and Xanthomonadaceae) and twelve bacterial genera (*Bacillus*, *Lysinibacillus*, *Metabacillus*, *Peribacillus*,* Brevibacillus*, *Staphylococcus, Acinetobacter, Psychrobacter*, *Pseudomonas, Pseudoxanthomonas, Xanthomonas* and *Stenotrophomonas*).

**Figure 5:**
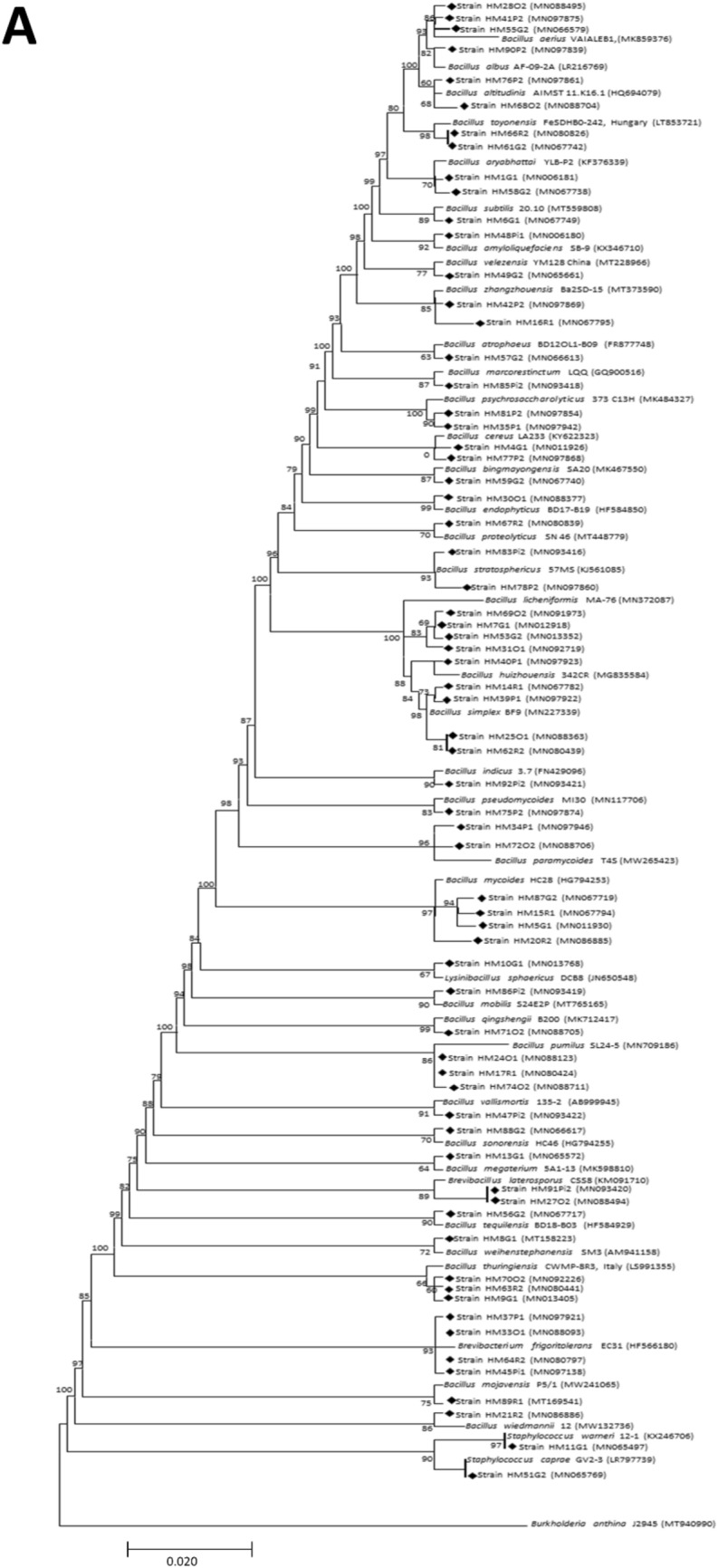

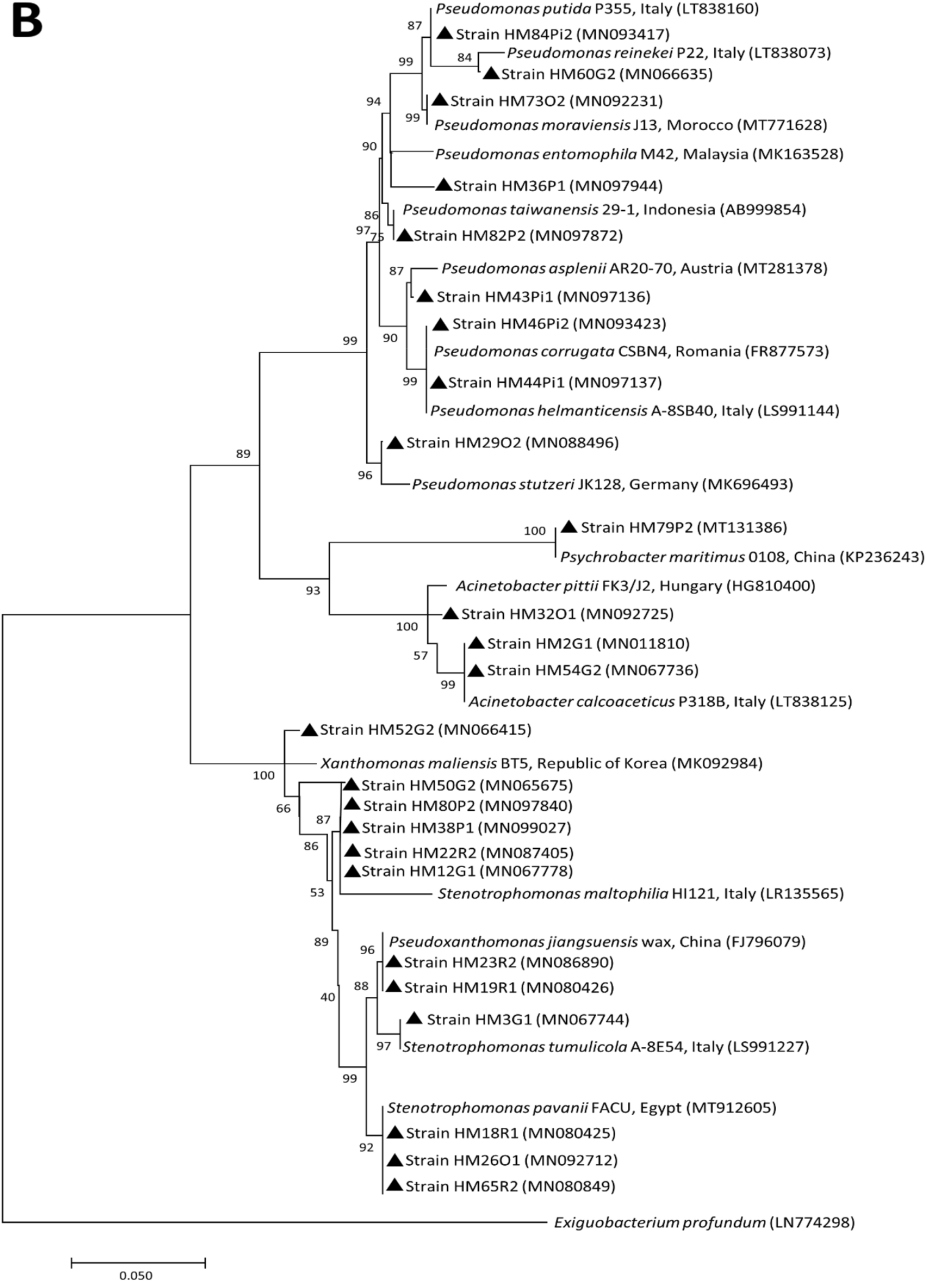
Phylogenetic trees depicting evolutionary relationship of isolated bacterial species from the phylum, Firmicutes **(A) **and Proteobacteria **(B)** based on their 16S rRNA gene sequences*.*

The evolutionary phylogenetic relationships based on 16S rRNA gene sequences of the bacterial species isolated from all of the study sites belonging to phylum, Firmicutes and Proteobacteria are given in the respective figures (Fig. 5A,B).

### Assessment of soil bacterial diversity in lower DNP

The diversity of the bacteria (Table 5) isolated from ten selected study sites covering five different micro- vegetational habitats of the lower DNP was determined by calculating the diversity indices such as Shannon–Wiener’s index (H′), Simpson’s index (d′), Dominance index (D), and Evenness index (*J*). The Shannon–Wiener’s index (H′) ranged from 1.380 to 2.631 with the lowest observed at site-9 (HAMP) whereas the highest was recorded at site-2 (LAG). The calculated values for Simpson’s diversity (d′) revealed it to be in the range of 0.749 to 0.923 with the lowest and highest values recorded from site-9 and site-2 respectively. On the other hand, the Dominance index (D) was found to be in the range of 0.078 to 0.253 with the lowest recorded value for site-2 and the highest for site-9 respectively. Likewise, the Evenness index (*J*) values of bacterial diversity showed the highest recorded value of 0.994 for site-9 however, the lowest value (0.920) did not depict to follow the same trend as it was observed for site-1 (HAG).

**Table 5:** Diversity of isolated bacterial strains from different study sites of Dachigam National Park, Kashmir.

Diversity index	HAG	LAG	HAR	LAR	HAO	LAO	HAP	LAP	HAMP	LAMP
Shannon (*H′*)	2.482	2.631	1.892	2.222	1.930	2.264	1.927	2.340	1.380	2.071
Simpson (*d′*)	0.910	0.923	0.840	0.887	0.852	0.893	0.852	0.899	0.747	0.873
Dominance (*D*)	0.090	0.078	0.160	0.113	0.148	0.107	0.148	0.101	0.253	0.127
Evenness (*J*)	0.920	0.926	0.947	0.923	0.984	0.963	0.981	0.944	0.994	0.991

The assessment of the soil bacterial species diversity in lower DNP ascertained that while some of the species were specific to a particular vegetation type, some others were present across various vegetation types although with differing morphological characteristics on account of being different strains of the same bacterial species (Table S4). During this study, among the fifty-seven (57) distinct bacterial strains, certain species namely *B. aerius, B. licheniformis, B. mycoides, B. pumilus, B. simplex, B. thuringiensis, B. frigoritolerans,* and *S. pavanii* showed a wider prevalence in the soils of DNP as they were found in three (03) or more sites whereas the bacteria namely *S. maltophilia,* was the most widespread as it was recorded from five out of the ten sites studied*.* However, the results clearly ascertained that about thirty-seven (37) species of bacteria identified in the present study, were found to be restricted to a particular site considering the differences in the vegetation as well as altitude at each site.

Thus, the similarity index (Table 6) of the bacteria isolated from the soils of ten study sites falling under five different vegetational types (i.e., grassland, riverine, oak, parrotiopsis and mixed pine) which were located at respective high and low altitude turned out to be—35.7% between grassland sites (HAG, site-1 and LAG, site-2); 47.1% between riverine sites (HAR, site-3 and LAR, site-4); 23.6% between oak sites (HAO, site-5 and LAO, site-6); and 22.2% between parrotiopsis sites, (HAP, site-7 and LAP, site-8). However, no similarity was found between the two mixed pine sites i.e., HAMP (site-9) and LAMP (site-10).

**Table 6:** Similarity index (%) of soil bacteria isolated from selected study sites falling under five vegetational types of lower Dachigam National Park, Kashmir.

Vegetation type	Sites	LAG	HAR	LAR	HAO	LAO	HAP	LAP	HAMP	LAMP
Grassland	HAG	35.7	10.0	26.1	10.0	17.4	10.0	16.7	0	0
	LAG		9.1	24.0	9.1	16.0	8.7	15.4	0	0
Riverine	HAR			47.1	42.9	11.8	14.3	11.1	0	0
	LAR				35.3	10.0	35.3	9.5	14.3	0
Oak	HAO					23.6	28.6	0	18.2	0
	LAO						11.8	19.1	0	11.1
Parrotiopsis	HAP							22.2	18.1	0
	LAP								0	10.5
Mixed pine	HAMP									0

## Discussion

National parks are key for the conservation of biodiversity by providing a safe haven for the threatened species to flourish and survive especially the endangered or endemic species^71^. DNP located in Zabarwan mountain range of western Himalaya is a habitat of great ecological significance as it is famous for harboring peculiar plant and animal forms comprising of more than 660 species of vascular plants, animals like critically endangered Kashmir red stag, Himalayan black bear, Himalayan brown bear, Himalayan yellow-throated marten, Himalayan gray langur, musk deer, common leopard, and about 150 bird species^53^. Thus, several researchers have extensively studied the national park for its phytodiversity and soil physicochemical characteristics^54,72^, its endangered animals^55,56,73^, but the national park has not been studied for its microbial populations including its bacteria in soil.

Soil is a chief component of the environment performing several vital functions as a result of which there is a continuous circulation of nutrients between various abiotic, and biotic processes. The temperature of soil influences its several operations (physical, chemical and biological) and is among the primary factors influencing such soil properties and processes which are engaged in the bacterial growth and developmental activities^74,75^. During the present study, soil temperature displayed significant (p = < 0.0001) differences between the study sites which can be attributed to the variations in the incoming radiation and the energy differences via the surface of the soil^76^, as these depend on factors like vegetation cover^77^, organic material content^78^, evaporation^79^, and inclination of land surface^80^. The soil moisture content in this study did not reflect any statistically significant differences (p = 0.11) among different sites which could be attributed to the depth differences from which the woody vegetation and grasses may obtain their soil water^81^, and variations in vegetation cover^82,83^.

In this research work, the maximum pH value (alkaline) was observed during winters whereas the lowest (slightly acidic) values were documented during the summer seasons which could be attributed to the presence of higher humus content releasing several acids in the soils^84^. Seasonal mean percentage of SOC and SOM showed a similar significant trend (p = < 0.0001). This could be either to the fine extensive root length of grasses per unit volume of soil, which is twenty times more in temperate grasslands in comparison to forest soils^85^, ratio of root to shoot which is about thirty times more in grasslands as compared to forests^86^, and enhanced process of organic matter decomposition^87^. The results of soil nitrogen suggested that the increase in soil nitrogen may be due to the higher amounts of soil organic matter^88–91^ and decreased N-mineralization as well as nitrification rates in forest soils with increase in altitude^92^ reflecting towards the temperature being the regulating factor.

Colony forming unit (CFU), which is an estimation of viable cells in a sample were determined for the seasonally collected soil samples over the period of 2 years (Spring, 2017–Winter, 2018). The results of CFU estimation revealed significant seasonal variations between the sites which can be attributed to the differences in general properties of soil, physico-chemical conditions and vegetation types as they are the major factors influencing the density, diversity, growth, and population of microorganisms including bacteria in soil^93,94^. Morphologically, in soil there is a prevalence of rod-shaped bacteria or bacilli followed by round/spherical-shaped bacteria or cocci and a few forms of spirillum-shaped bacteria or spirilla^95^ which are in accordance to the findings of this work. As has been documented here, earlier studies have also reported the pre-dominance of Gram-positive in comparison to Gram-negative bacterial forms obtained from diverse soils^96–98^.

The diversity of the culturable bacteria in different altitudinally varied micro-vegetational habitats of DNP revealed clear variations with respect to differences in soil properties as well as vegetation types which are often considered to be the vital factors affecting the density, diversity, growth and populations of bacteria in soil^31,93,94,99^. The vegetational diversity is considered to likely affect the soil bacterial activity, biomass as well as their composition, either in a direct manner by the production of litter and root exudates, or in an indirect way by influencing and changing the physico-chemical properties of soil^100–102^. In our study, results depicted that in Grassland vegetation, there was pre-dominance of soil bacterial species belonging to Firmicutes which are capable of surviving under harsh environments^103,104^. Lugo et al.^105^ in their study, reported species of genera *Arthrobacter*, *Bacillus,* and *Pseudomonas*, during their investigation on the rhizospheric bacterial diversity in a South-American grassland whereas from the Grasslands under consideration, we reported more diverse bacterial species belonging to genus, *Acinetobacter, Bacillus, Lysinibacillus, Staphylococcus, Stenotrophomonas, Xanthomonas, Pseudomonas* with the dominance of *Bacillus* species.

Riverine forests mostly comprise of moist temperate deciduous broad-leaved trees producing relatively higher quantities of litter and have enhanced decomposition rates in comparison to coniferous trees, which result in higher nutrient levels supporting more dense and diverse microbial populations^106^ and a similar trend has been noticed in the current study whereby the total bacterial species at Riverine vegetational sites were more than what has been recorded in Mixed Pine sites. Microbial communities have been found to be distinct not only between the forest and pasture soils^107^ but also among various forests^108^. Chim Chan et al.^109^ while studying the impact of vegetational covers (forests, shrubs, pastures) on the soil bacteria, concluded that while Acidobacteria dominated the broad-leaf forests, Proteobacteria and Firmicutes was prevalent in the soils of shrub and the pastures showed the predominance of alpha- and beta-Proteobacteria and Bacteroidetes. A similar observation was made in our study whereby the bacterial species under shrub vegetational type (Parrotiopsis) were mainly found to be comprised of Firmicutes (13) and Proteobacteria (05). During the present investigation, as far as the number of bacterial species in the Mixed Pine vegetational sites are concerned, the results clearly showed that among all vegetational types, the least number of bacteria were recorded from these sites. This could be attributed to the quality of the litter as well as its decomposition rates as several studies on the conifers have suggested that the litter of the coniferous tree species is rich in the amounts of acids, lignin, tannins, and other phenolic compounds, thereby making its decomposition arduous as a result of which there is a strong influence on the growth of soil microorganisms^110–112^.

As various ecological factors are considered to affect the diversity and distribution of bacteria in soil^113^, several studies have concluded that the regions situated at higher altitudes possess scanty vegetation constituting distinctive soil attributes which support less diverse bacterial community and structure in comparison to those regions located at lower ones supporting higher bacterial diversity^31,114^ which is in agreement with the findings of this study. Estimates have predicted that the values of the Shannon–Wiener’s Index (H′) typically range from 1.5 to 3.5 for ecological data^115^, and in this study, the H′-value ranged from > 1.3 up to as high as > 2.6 which clearly reflect the prevalence of bacterial diversity in lower DNP. On the other hand, the evenness index (J) of the bacteria in soil reflects certain pressures that might shape their community diversity and thus, its measurement is considered to be among one of the most significant attributes while assessing the impacts of various environmental factors on the diversity of bacteria in soil^116^. Evenness index (J) in this study didn’t reflect any significant trend within the sites which are consistent with the findings of Bryant et al.^117^, Fierer et al.^118^ and Lyngwi et al.^31^. Furthermore, the variations in the composition of vegetation displayed significant influence on the similarity index of the soil bacterial populations among the five studied vegetational types wherein the highest similarity (> 47%) was depicted among the Riverine vegetational sites and no similarity was recorded in between the Mixed Pine vegetational sites. This is in accordance with the study of Liu et al.^99^ which concluded that tree species compositions had a profound influence on the similarity coefficients of their rhizospheric soil bacterial communities.

## Conclusions

The north-western part of Himalayas encompassing the Zabarwan range of Kashmir is known to consist different climatic and elevational zones having varied soil textures which serves as an access-point inhabiting the richest bio-diversity with great level of endemism. However, this region is not extensively studied as far as its microbial diversity including bacteria is concerned. In this study, the physico-chemical parameters of the soil were determined, the diversity and distribution of culturable soil bacteria from low to high (1671–1870 m.a.s.l.) elevational gradient spreading across five different vegetation types were characterized, and correlated with the soil bacterial distribution and diversity. Soil parameters such as temperature, pH, moisture content, organic carbon and matter, and nitrogen were measured seasonally at each site. The bacteria in soil were cultured on three different media (Nutrient agar, Luria Bertani agar and Reasoner’s 2A agar) and were initially characterized by morphological and Gram staining methods. A total of 92 morphologically different bacterial isolates were then subjected to 16S rRNA sequence analysis for estimating the diversity of lower DNP. The phylogenetic analysis of these 92 isolated strains varying in their macro- and micro-morphological characteristics, revealed the presence of only fifty-seven (57) different species at the molecular level with the Firmicutes being the most common bacterial group, followed by Proteobacteria. Bacterial CFUs showed a positive correlation with parameters of the soil, such as temperature (r = 0.66), organic carbon content (r = 0.55), organic matter content (r = 0.55), and total nitrogen content (r = 0.5), whereas moisture content (r = − 0.12) and pH (r = − 0.4) of soil showed a negative correlation. The results of this study clearly reflected that the altitudinal gradient, coupled with the varied vegetational types and the soil physico-chemical parameters influenced the distribution and diversity of bacteria in soil. This study therefore concludes that lower DNP, an ecologically significant biome contains a vast reservoir of soil bacteria which decreases with increasing altitude and thus provide us with the first baseline information highlighting the microbial importance from this poorly explored area in the western Himalaya, with justifying efforts for the presence and need to explore the prevalence of novel species in this vital ecosystem.

## Supplementary Information

Supplementary Information.

## Data Availability

Data regarding 16S rRNA gene sequences have been deposited in GenBank, NCBI (https://www.ncbi.nlm.nih.gov/nucleotide/) under the accession numbers given in the table (Table S3) contained in Supplementary Information.
