# Supplementary material for: Exploring soil bacterial diversity in different micro-vegetational habitats of Dachigam National Park in North-western Himalaya
Source: Sci Rep. 2023 Feb 22;13:3090. doi: 10.1038/s41598-023-30187-w (PMC9947166; doi:10.1038/s41598-023-30187-w)
Supplement: Supplementary file 1 — Supplementary Information. [file 41598_2023_30187_MOESM1_ESM.pdf]

Exploring soil bacterial diversity in different micro-vegetational habitats of Dachigam National Park in North-western Himalaya

Hina Mushtaq[1], Bashir Ahmad Ganai[2], and Arshid Jehangir[1*]

[1]Department of Environmental Science, University of Kashmir, Hazratbal, Srinagar-190006, Jammu and Kashmir, India.

[2]Centre of Research for Development, University of Kashmir, Hazratbal, Srinagar-190006, Jammu and Kashmir, India.

***Corresponding Author**

Dr. Arshid Jehangir
Senior Assistant Professor
Terrestrial Ecology Laboratory
Department of Environmental Science
University of Kashmir, Hazratbal (Srinagar),
Jammu and Kashmir (India)
E-mail: arshidj@gmail.com
Contact number: +91-9797070540

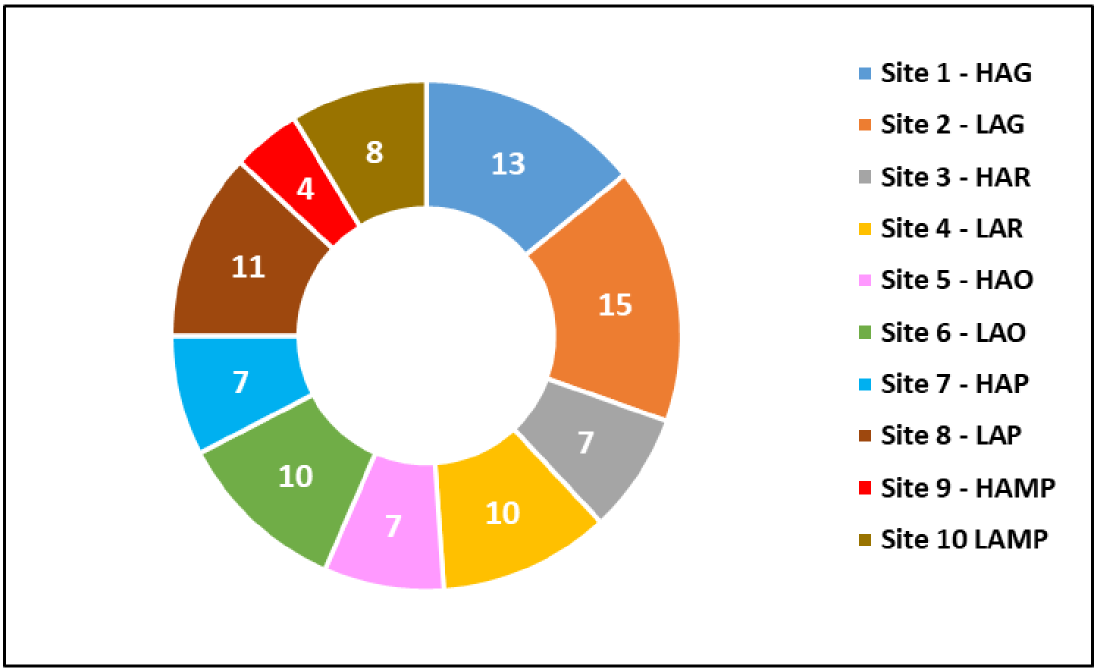

Figure S1A. Site-wise number of bacterial strains isolated from the soils of selected vegetational microhabitat in lower DNP

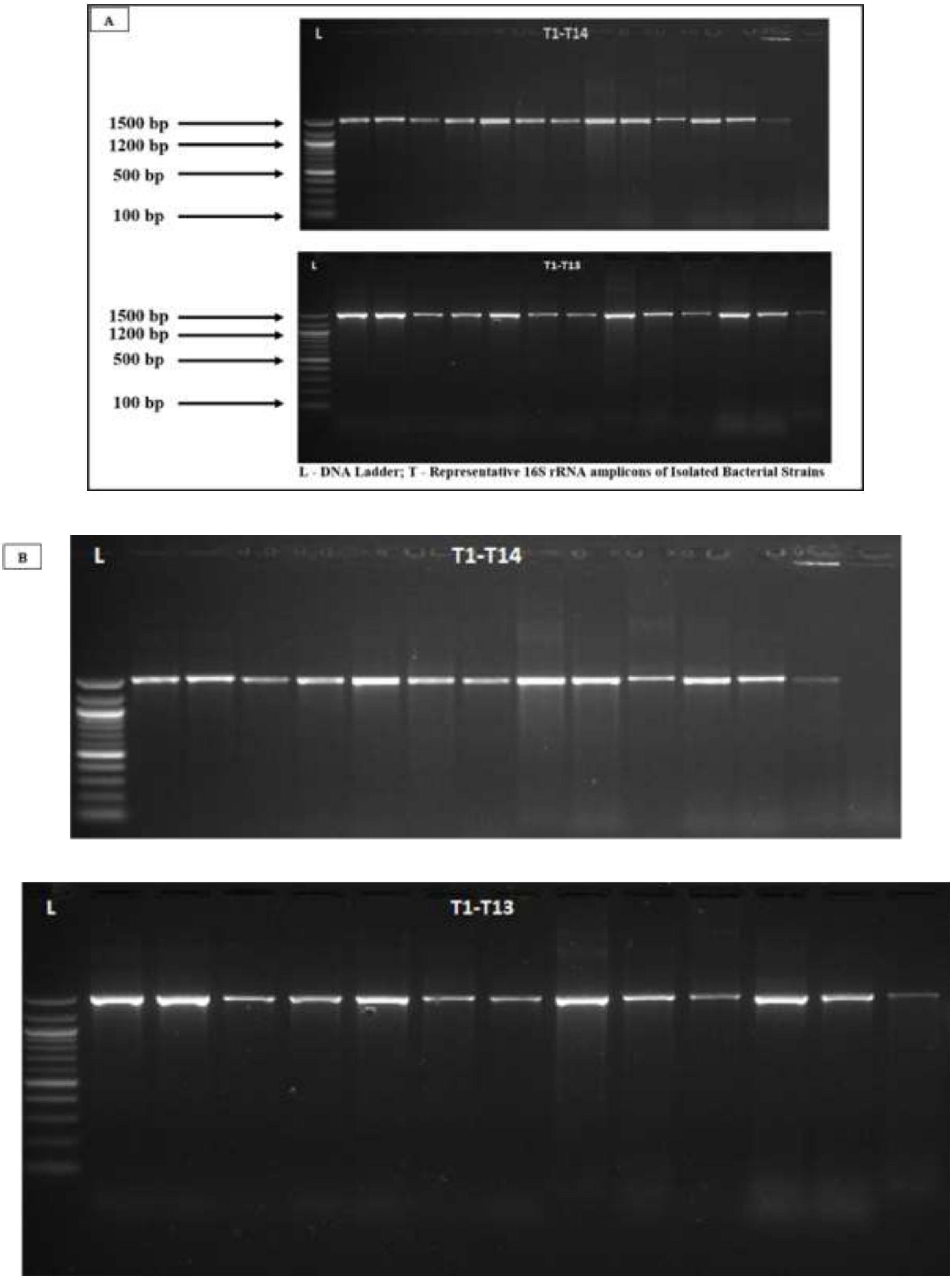

Figure S1B. (A) Representative electrophoretic images of the amplified PCR products of 16S rRNA gene of the isolated bacterial strains from lower DNP (B) Original Images

Table S1. Macro-morphological colony characteristics and Gram-staining properties of the isolated bacterial strains

Isolate ID	Size	Shape	Elevation	Texture	Appearance	Colour	Optical property	Gram's Reaction	Cell Shape
**HM1G1**	Moderate	Circular	Flat	Rough	Shiny	Cream	Translucent	+	Bacilli
**HM2G1**	Moderate	Circular	Convex	Smooth	Shiny	Cream	Opaque	-	Cocci
**HM3G1**	Moderate	Circular	Flat	Smooth	Dull	Cream	Opaque	-	Bacilli
**HM4G1**	Moderate	Circular	Convex	Smooth	Shiny	Yellow	Opaque	+	Bacilli
**HM5G1**	Moderate	Circular	Raised	Rough	Dull	Red	Opaque	+	Bacilli
**HM6G1**	Punctiform	Irregular	Flat	Rough	Dull	Cream	Translucent	+	Bacilli
**HM7G1**	Moderate	Circular	Raised	Smooth	Shiny	Cream	Translucent	+	Bacilli
**HM8G1**	Large	Circular	Raised	Rough	Dull	Cream	Opaque	+	Bacilli
**HM9G1**	Large	Circular	Convex	Smooth	Dull	Cream	Opaque	+	Bacilli
**HM10G1**	Moderate	Circular	Raised	Smooth	Shiny	Cream	Opaque	+	Bacilli
**HM11G1**	Small	Circular	Raised	Smooth	Shiny	Cream	Opaque	+	Cocci
**HM12G1**	Moderate	Circular	Convex	Rough	Shiny	Cream	Translucent	-	Bacilli
**HM13G1**	Moderate	Circular	Raised	Rough	Dull	Cream	Opaque	+	Bacilli
**HM14R1**	Moderate	Circular	Convex	Rough	Shiny	Cream	Opaque	+	Bacilli
**HM15R1**	Large	Rhizoid	Flat	Rough	Shiny	Cream	Translucent	+	Bacilli
**HM16R1**	Moderate	Circular	Flat	Rough	Dull	Cream	Opaque	+	Bacilli
**HM17R1**	Small	Circular	Convex	Smooth	Shiny	Yellow	Translucent	+	Bacilli
**HM18R1**	Moderate	Circular	Raised	Rough	Shiny	Tan	Opaque	-	Bacilli
**HM19R1**	Moderate	Circular	Raised	Rough	Shiny	Tan	Opaque	-	Bacilli
**HM20R2**	Punctiform	Circular	Convex	Smooth	Shiny	Yellow	Translucent	-	Cocci
**HM21R2**	Moderate	Circular	Raised	Smooth	Shiny	Tan	Transparent	+	Bacilli
**HM22R2**	Moderate	Circular	Raised	Smooth	Shiny	Tan	Transparent	-	Bacilli
**HM23R2**	Small	Circular	Raised	Rough	Shiny	Cream	Opaque	-	Bacilli
**HM24O1**	Large	Circular	Raised	Rough	Shiny	Cream	Opaque	+	Bacilli
**HM25O1**	Large	Circular	Convex	Smooth	Shiny	Cream	Opaque	+	Bacilli
**HM26O1**	Small	Circular	Convex	Smooth	Shiny	Tan	Translucent	-	Bacilli
**HM27O2**	Large	Irregular	Flat	Smooth	Dull	Cream	Opaque	+	Bacilli
**HM28O2**	Large	Circular	Convex	Smooth	Shiny	Cream	Opaque	+	Bacilli
**HM29O2**	Moderate	Circular	Umbonate	Rough	Dull	Tan	Translucent	-	Bacilli
**HM30O1**	Small	Circular	Convex	Rough	Shiny	Tan	Translucent	+	Bacilli
**HM31O1**	Punctiform	Circular	Raised	Smooth	Shiny	Tan	Opaque	+	Bacilli

Table S1. Macro-morphological colony characteristics and Gram-staining properties of the isolated bacterial strains

Isolate ID	Size	Shape	Elevation	Texture	Appearance	Colour	Optical property	Gram's Reaction	Cell Shape
**HM32O1**	Large	Irregular	Raised	Smooth	Shiny	Red	Opaque	-	Cocci
**HM33O1**	Moderate	Circular	Pulvinate	Smooth	Shiny	Cream	Opaque	+	Bacilli
**HM34P1**	Large	Rhizoid	Flat	Rough	Dull	White	Translucent	+	Bacilli
**HM35P1**	Large	Circular	Convex	Smooth	Shiny	Cream	Opaque	+	Bacilli
**HM36P1**	Large	Irregular	Raised	Smooth	Shiny	Cream	Opaque	-	Bacilli
**HM37P1**	Large	Circular	Convex	Smooth	Shiny	Cream	Translucent	+	Cocci
**HM38P1**	Moderate	Circular	Raised	Smooth	Dull	Cream	Opaque	-	Bacilli
**HM39P1**	Moderate	Circular	Raised	Smooth	Shiny	Cream	Opaque	+	Bacilli
**HM40P1**	Moderate	Circular	Raised	Smooth	Shiny	White	Opaque	+	Bacilli
**HM41P2**	Large	Circular	Raised	Smooth	Shiny	Tan	Translucent	+	Bacilli
**HM42P2**	Large	Circular	Pulvinate	Smooth	Shiny	Cream	Translucent	+	Bacilli
**HM43Pi1**	Moderate	Circular	Raised	Smooth	Shiny	Tan	Translucent	-	Bacilli
**HM44Pi1**	Small	Circular	Raised	Rough	Shiny	Cream	Translucent	-	Bacilli
**HM45Pi1**	Moderate	Circular	Raised	Rough	Dull	Cream	Opaque	+	Cocci
**HM46Pi2**	Small	Circular	Raised	Smooth	Shiny	Cream	Opaque	-	Bacilli
**HM47Pi2**	Moderate	Circular	Raised	Rough	Dull	Cream	Opaque	-	Bacilli
**HM48Pi1**	Moderate	Circular	Raised	Rough	Dull	Cream	Opaque	+	Bacilli
**HM49G2**	Moderate	Circular	Flat	Rough	Dull	Cream	Opaque	+	Bacilli
**HM50G2**	Large	Circular	Umbonate	Smooth	Shiny	Yellow	Translucent	-	Bacilli
**HM51G2**	Moderate	Circular	Convex	Smooth	Shiny	Yellow	Opaque	+	Cocci
**HM52G2**	Moderate	Circular	Convex	Smooth	Shiny	Cream	Opaque	-	Bacilli
**HM53G2**	Small	Circular	Umbonate	Smooth	Shiny	Cream	Translucent	+	Bacilli
**HM54G2**	Large	Circular	Pulvinate	Smooth	Shiny	Cream	Translucent	-	Cocci
**HM55G2**	Moderate	Circular	Pulvinate	Smooth	Shiny	Cream	Opaque	+	Bacilli
**HM56G2**	Small	Circular	Convex	Smooth	Shiny	Yellow	Opaque	+	Bacilli
**HM57G2**	Large	Irregular	Raised	Rough	Dull	Cream	Opaque	+	Bacilli
**HM58G2**	Small	Circular	Convex	Rough	Shiny	Yellow	Opaque	+	Bacilli
**HM59G2**	Small	Circular	Raised	Smooth	Shiny	Cream	Translucent	+	Bacilli
**HM60G2**	Moderate	Circular	Raised	Rough	Shiny	Tan	Transparent	-	Bacilli
**HM61G2**	Large	Circular	Raised	Rough	Dull	Tan	Translucent	+	Bacilli
**HM62R2**	Large	Circular	Raised	Rough	Shiny	Cream	Opaque	+	Bacilli

Table S1. Macro-morphological colony characteristics and Gram-staining properties of the isolated bacterial strains

Isolate ID	Size	Shape	Elevation	Texture	Appearance	Colour	Optical property	Gram's Reaction	Cell Shape
**HM63R2**	Large	Circular	Flat	Rough	Dull	Cream	Opaque	+	Cocci
**HM64R2**	Large	Circular	Raised	Rough	Dull	Cream	Opaque	+	Cocci
**HM65R2**	Moderate	Circular	Convex	Smooth	Shiny	Cream	Opaque	-	Bacilli
**HM66R2**	Moderate	Circular	Convex	Rough	Dull	Cream	Opaque	+	Bacilli
**HM67R2**	Moderate	Circular	Raised	Smooth	Dull	Cream	Opaque	+	Bacilli
**HM68O2**	Small	Circular	Convex	Smooth	Shiny	Cream	Translucent	+	Bacilli
**HM69O2**	Small	Circular	Raised	Rough	Dull	Cream	Opaque	+	Bacilli
**HM70O2**	Small	Circular	Convex	Smooth	Shiny	Cream	Opaque	+	Bacilli
**HM71O2**	Small	Circular	Umbonate	Rough	Dull	Cream	Opaque	+	Bacilli
**HM72O2**	Moderate	Circular	Raised	Smooth	Shiny	Cream	Opaque	+	Bacilli
**HM73O2**	Punctiform	Circular	Convex	Smooth	Shiny	Cream	Opaque	-	Bacilli
**HM74O2**	Moderate	Circular	Raised	Rough	Dull	Cream	Opaque	+	Bacilli
**HM75P2**	Large	Rhizoid	Flat	Rough	Dull	Cream	Opaque	+	Bacilli
**HM76P2**	Moderate	Circular	Flat	Smooth	Dull	Cream	Translucent	+	Bacilli
**HM77P2**	Moderate	Circular	Raised	Smooth	Shiny	Tan	Translucent	+	Bacilli
**HM78P2**	Large	Circular	Flat	Rough	Dull	Cream	Translucent	+	Bacilli
**HM79P2**	Moderate	Circular	Convex	Smooth	Shiny	Cream	Translucent	-	Cocci
**HM80P2**	Moderate	Circular	Convex	Smooth	Shiny	Tan	Translucent	-	Bacilli
**HM81P2**	Moderate	Circular	Pulvinate	Rough	Dull	Cream	Opaque	+	Bacilli
**HM82P2**	Small	Circular	Convex	Rough	Dull	Cream	Opaque	-	Bacilli
**HM83Pi2**	Punctiform	Circular	Raised	Smooth	Shiny	Tan	Opaque	+	Bacilli
**HM84Pi2**	Moderate	Circular	Raised	Smooth	Shiny	Cream	Opaque	-	Bacilli
**HM85Pi2**	Large	Rhizoid	Flat	Rough	Dull	Cream	Opaque	+	Bacilli
**HM86Pi2**	Moderate	Circular	Convex	Smooth	Shiny	Yellow	Opaque	+	Bacilli
**HM87G2**	Large	Filamentous	Raised	Rough	Dull	Cream	Opaque	+	Bacilli
**HM88G2**	Large	Circular	Umbonate	Rough	Dull	Cream	Translucent	+	Bacilli
**HM89R1**	Moderate	Circular	Raised	Smooth	Shiny	Yellow	Opaque	+	Bacilli
**HM90P2**	Large	Rhizoid	Flat	Rough	Dull	Cream	Opaque	+	Bacilli
**HM91Pi2**	Moderate	Irregular	Flat	Smooth	Dull	Cream	Opaque	+	Bacilli
**HM92Pi2**	Moderate	Circular	Convex	Smooth	Shiny	Yellow	Opaque	+	Bacilli

Table S2. Percentage occurrence of Gram-positive and Gram-negative bacteria isolated from different vegetational soils of lower DNP

Gram's Reaction	Shape	Number (Out of total, 92)	Percentage (%)	Total Number	Total Percentage (%)
Gram Positive	Bacilli	59	64.13	65	70.65
	Cocci	06	6.52		
Gram Negative	Bacilli	22	23.91	27	29.35
	Cocci	05	5.43		

Table S3. Molecular identification of the isolated bacterial strains

S.No.	Isolate ID	Species	Identities (%)	GenBank Accession Number
1.	**HM1G1**	*Bacillus aryabhattai*	97	MN006181
2.	**HM2G1**	*Acinetobacter calcoaceticus*	98	MN011810
3.	**HM3G1**	*Stenotrophomonas tumulicola*	96	MN067744
4.	**HM4G1**	*Bacillus cereus*	95	MN011926
5.	**HM5G1**	*Bacillus mycoides*	99	MN011930
6.	**HM6G1**	*Bacillus subtilis*	99	MN067749
7.	**HM7G1**	*Bacillus licheniformis*	93	MN012918
8.	**HM8G1**	*Bacillus weihenstephanensis*	100	MT158223
9.	**HM9G1**	*Bacillus thuringiensis*	98	MN013405
10.	**HM10G1**	*Lysinibacillus sphaericus*	99	MN013768
11.	**HM11G1**	*Staphylococcus warneri*	93	MN065497
12.	**HM12G1**	*Stenotrophomonas maltophilia*	98	MN067778
13.	**HM13G1**	*Bacillus megaterium*	99	MN065572
14.	**HM14R1**	*Bacillus simplex*	99	MN067782
15.	**HM15R1**	*Bacillus mycoides*	100	MN067794
16.	**HM16R1**	*Bacillus zhangzhouensis*	100	MN067795
17.	**HM17R1**	*Bacillus pumilus*	100	MN080424
18.	**HM18R1**	*Stenotrophomonas pavanii*	97	MN080425
19.	**HM19R1**	*Pseudoxanthomonas jiangsuensis*	95	MN080426
20.	**HM20R2**	*Bacillus mycoides*	99	MN086885
21.	**HM21R2**	*Bacillus wiedmannii*	99	MN086886
22.	**HM22R2**	*Stenotrophomonas maltophilia*	97	MN08705
23.	**HM23R2**	*Pseudoxanthomonas jiangsuensis*	95	MN086890
24.	**HM24O1**	*Bacillus pumilus*	98	MN088213
25.	**HM25O1**	*Bacillus simplex*	99	MN088363
26.	**HM26O1**	*Stenotrophomonas pavanii*	97	MN092712
27.	**HM27O2**	*Brevibacillus laterosporus*	95	MN088494
28.	**HM28O2**	*Bacillus aerius*	99	MN088495
29.	**HM29O2**	*Pseudomonas stutzeri*	99	MN088496
30.	**HM30O1**	*Bacillus endophyticus*	99	MN088377
31.	**HM31O1**	*Bacillus licheniformis*	95	MN092719
32.	**HM32O1**	*Acinetobacter pittii*	97	MN092725
33.	**HM33O1**	*Brevibacterium frigoritolerans*	100	MN088093
34.	**HM34P1**	*Bacillus paramycoides*	99	MN097946
35.	**HM35P1**	*Bacillus psychrosaccharolyticus*	98	MN097942
36.	**HM36P1**	*Pseudomonas entomophila*	100	MN097944
37.	**HM37P1**	*Brevibacterium frigoritolerans*	99	MN097921
38.	**HM38P1**	*Stenotrophomonas maltophilia*	97	MN099027
39.	**HM39P1**	*Bacillus simplex*	96	MN097922
40.	**HM40P1**	*Bacillus huizhouensis*	95	MN097923
41.	**HM41P2**	*Bacillus aerius*	100	MN097875
42.	**HM42P2**	*Bacillus zhangzhouensis*	99	MN097869
43.	**HM43Pi1**	*Pseudomonas asplenii*	99	MN097136
44.	**HM44Pi1**	*Pseudomonas helmanticensis*	98	MN097137
45.	**HM45Pi1**	*Brevibacterium frigoritolerans*	99	MN097138
46.	**HM46Pi2**	*Pseudomonas corrugata*	99	MN093423

Table S3. Molecular identification of the isolated bacterial strains

S.No.	Isolate ID	Species	Identities (%)	GenBank Accession Number
47.	**HM47Pi2**	*Bacillus vallismortis*	99	MN093422
48.	**HM48Pi1**	*Bacillus amyloliquefaciens*	99	MN006180
49.	**HM49G2**	*Bacillus velezensis*	99	MN065661
50.	**HM50G2**	*Stenotrophomonas maltophilia*	98	MN065675
51.	**HM51G2**	*Staphylococcus caprae*	99	MN065769
52.	**HM52G2**	*Xanthomonas maliensis*	95	MN066415
53.	**HM53G2**	*Bacillus licheniformis*	95	MN013352
54.	**HM54G2**	*Acinetobacter calcoaceticus*	98	MN067736
55.	**HM55G2**	*Bacillus aerius*	99	MN066579
56.	**HM56G2**	*Bacillus tequilensis*	99	MN067717
57.	**HM57G2**	*Bacillus atrophaeus*	99	MN066613
58.	**HM58G2**	*Bacillus aryabhattai*	97	MN067738
59.	**HM59G2**	*Bacillus bingmayongensis*	99	MN067740
60.	**HM60G2**	*Pseudomonas reinekei*	99	MN066635
61.	**HM61G2**	*Bacillus toyonensis*	93	MN067742
62.	**HM62R2**	*Bacillus simplex*	99	MN080439
63.	**HM63R2**	*Bacillus thuringiensis*	99	MN080441
64.	**HM64R2**	*Brevibacterium frigoritolerans*	99	MN080797
65.	**HM65R2**	*Stenotrophomonas pavanii*	97	MN080849
66.	**HM66R2**	*Bacillus toyonensis*	99	MN080826
67.	**HM67R2**	*Bacillus proteolyticus*	99	MN080839
68.	**HM68O2**	*Bacillus altitudinis*	99	MN088704
69.	**HM69O2**	*Bacillus licheniformis*	94	MN091973
70.	**HM70O2**	*Bacillus thuringiensis*	98	MN092226
71.	**HM71O2**	*Bacillus qingshengii*	99	MN088705
72.	**HM72O2**	*Bacillus paramycoides*	98	MN088706
73.	**HM73O2**	*Pseudomonas moraviensis*	99	MN092231
74.	**HM74O2**	*Bacillus pumilus*	97	MN088711
75.	**HM75P2**	*Bacillus pseudomycoides*	99	MN097874
76.	**HM76P2**	*Bacillus altitudinis*	96	MN097861
77.	**HM77P2**	*Bacillus cereus*	98	MN097868
78.	**HM78P2**	*Bacillus stratosphericus*	96	MN097860
79.	**HM79P2**	*Psychrobacter maritimus*	100	MT131386
80.	**HM80P2**	*Stenotrophomonas maltophilia*	97	MN097840
81.	**HM81P2**	*Bacillus psychrosaccharolyticus*	98	MN097854
82.	**HM82P2**	*Pseudomonas taiwanensis*	99	MN097872
83.	**HM83Pi2**	*Bacillus stratosphericus*	99	MN093416
84.	**HM84Pi2**	*Pseudomonas putida*	98	MN093417
85.	**HM85Pi2**	*Bacillus marcorestinctum*	97	MN093418
86.	**HM86Pi2**	*Bacillus mobilis*	99	MN093419
87.	**HM87G2**	*Bacillus mycoides*	100	MN067719
88.	**HM88G2**	*Bacillus sonorensis*	94	MN066617
89.	**HM89R1**	*Bacillus mojavensis*	100	MT169541
90.	**HM90P2**	*Bacillus albus*	97	MN097839
91.	**HM91Pi2**	*Brevibacillus laterosporus*	97	MN093420
92.	**HM92Pi2**	*Bacillus indicus*	100	MN093421

Table S4. Site-wise account of unique bacterial species isolated from soils of lower DNP

Site/Site code	Number of the Species (Out of total, 37)	Name of the Bacterial Species
Site-1 (HAG)	06	*B. megaterium*, *B. subtilis*, *B. weihenstephanensis*, *L. sphaericus*, *S. warneri*, and *S. tumulicola*
Site-2 (LAG)	08	*B. atrophaeus*, *B. bingmayongensis*, *B. sonorensis*, *B. tequilensis*, *B. velezensis*, *P. reinekei*, *S. caprae*, and *X. maliensis*
Site-3 (HAR)	01	*B. mojavensis*
Site-4 (LAR)	02	*B. proteolyticus* and *B. wiedmannii*
Site-5 (HAO)	02	*A. pittii*, and *B. endophyticus*
Site-6 (LAO)	03	*B. qingshengii*, *P. moraviensis* and *P. stutzeri*
Site-7 (HAP)	02	*B. huizhouensis* and *P. entomophila*
Site-8 (LAP)	04	*B. albus*, *B. pseudomycoides*, *P. taiwanensis* and *P. maritimus*
Site-9 (HAMP)	03	*B. amyloliquefaciens*, *P. asplenii*, and *P. helmanticensis*
Site-10 (LAMP)	06	*B. indicus*, *B. marcorestinctum*, *B. mobilis*, *B. vallismortis*, *P. corrugata*, and *P. putida*
